# Case report: Two cases of primary paratesticular liposarcoma

**DOI:** 10.3389/fonc.2022.1040458

**Published:** 2022-10-17

**Authors:** Jiaxing Li, Jiayu Wang, Hu Han, Long Tian, Hang Yin

**Affiliations:** ^1^ Department of Urology, Beijing Chao-Yang Hospital, Capital Medical University, Beijing, China; ^2^ Institute of Urology, Beijing Chao-Yang Hospital, Capital Medical University, Beijing, China

**Keywords:** testicular tumor, liposarcoma, paratesticular liposarcoma, dedifferentiated, scrotoscope

## Abstract

Paratesticular liposarcoma is a sporadic urological tumor. We report the clinical presentation, treatment course, and prognosis of 2 cases of primary paratesticular liposarcoma with different pathological types, with the aim of further understanding the diagnosis and treatment of this rare disease. Case 1 was a 53-years-old male patient who presented with left scrotal enlargement with swelling 3 years ago and was considered to have a testicular malignancy on preoperative CT scan and enhanced MRI. The patient underwent resection of the left scrotal mass and left orchiectomy under general anaesthesia. Histopathological study confirmed the diagnosis of dedifferentiated liposarcoma. At the 4-months follow-up, no local recurrence or distant metastasis was observed. Case 2 is a 42-years-old male patient with a left scrotal mass which was discovered six months ago. Preoperatively, he underwent CT plain and enhanced MRI examinations suggesting an intra-scrotal occupancy. The patient underwent scrotoscopic excision of the left scrotal mass under general anesthesia. Histopathological studies confirmed the diagnosis of highly differentiated liposarcoma. At the 10-months follow-up, no local recurrence or distant metastasis was observed. Preoperative differential diagnosis of paratesticular liposarcoma should be noted with testicular tumor and extra-abdominal hernia. Extensive local excision and, if necessary, concomitant radical testicular resection is the treatment of choice. If the tumor in the scrotum spreads to the inguinal region, surgical removal with the aid of a scrotoscope may be attempted. This procedure avoids the formation of a large incision in the inguinal region compared to traditional open surgery. Patients commonly experience local recurrence and less distant metastases after surgery, so long-term follow-up is recommended.

## Introduction:

Paratesticular liposarcoma (PLS) accounts for approximately 7% of the scrotal tumor. And it is a sporadic urologic tumor. Only about 265 cases have been reported worldwide (1). The 2020 World Health Organization (WHO) classification of soft tissue and bone tumors distinguishes four major liposarcomas (LPS) subtypes in the group of adipocytic tumors: atypical lipomatous tumor/well-differentiated LPS (ALT/WDLPS), dedifferentiated LPS (DDLPS), myxoid LPS and pleomorphic LPS. 80%~90% of DDLPS are primary, and 10% of cases can occur with secondary dedifferentiation (2). Mostly they locate in the deeper tissues of the retroperitoneum, extremities, and mediastinum and they are rare in the paratesticular region. PLS is more common in WDLPS and DDLPS. We describe the clinical history, treatment, and features of two cases of primary PLS, one is a giant paratesticular DDLPS and the other is a paratesticular WDLPS, with the aim of further understanding the diagnosis and treatment of this rare condition. Patients have provided their written informed consents for the publication of this manuscript and some identifying images or data.

## Case presentation:

### Case1:

#### Chief complaints and History of illness:

A 53-years-old male patient was admitted to the hospital with “an enlarged left scrotum with a feeling of swelling for more than 3 years”. The patient was previously healthy, had no smoking history, and did not consume alcohol. There was no history of disease or surgery. There was no specific family history, including cancer.

#### Physical examination:

**Figure 1:**
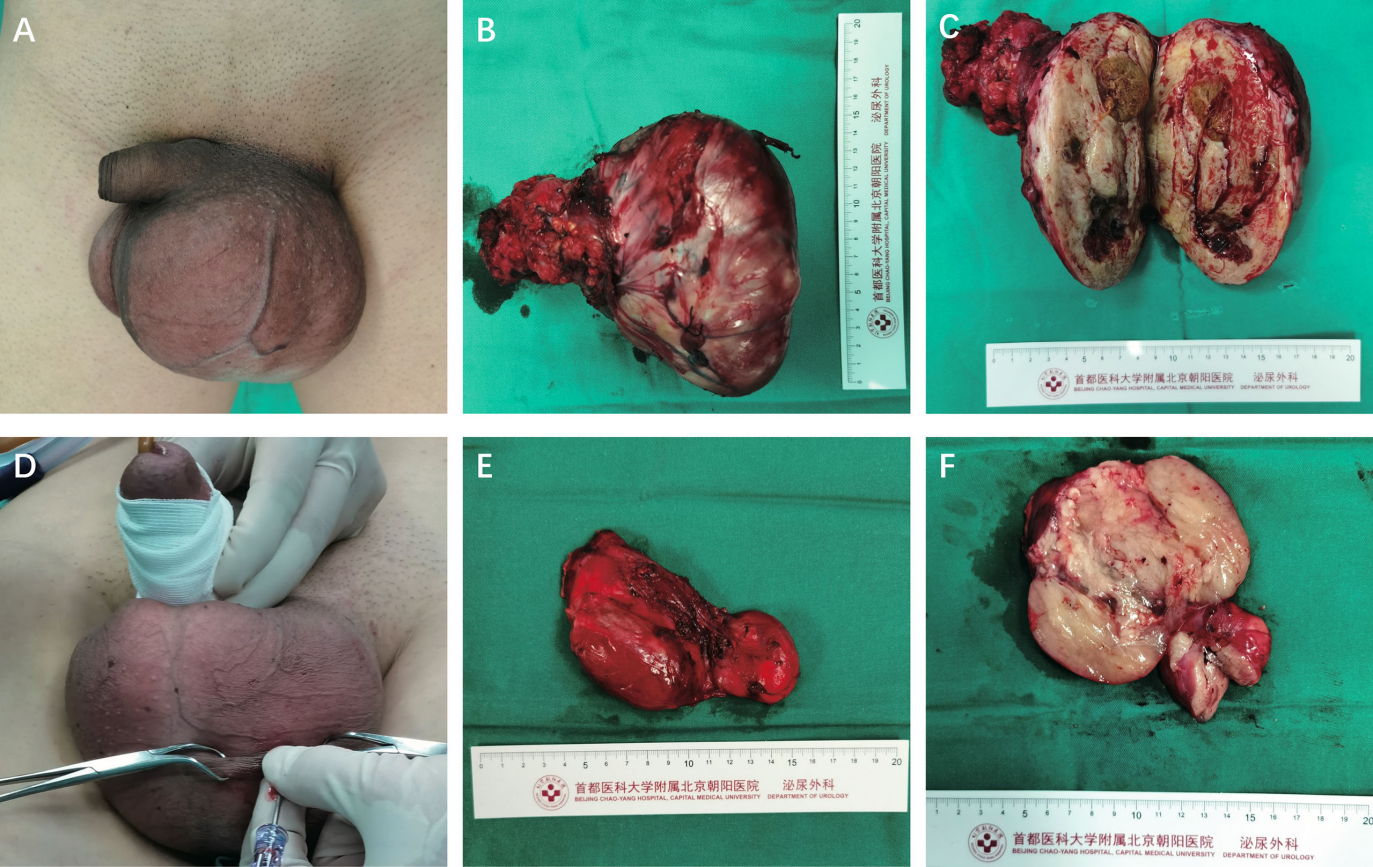
**(A)** Case 1 physical examination. **(B)** Gross pathologic examination revealed that the giant tumor was a grayish-white and grayish-yellow mass, measuring 15cm×9cm×9cm, and its capsule was intact. **(C)** The cut plane of the tumor was grayish white and grayish yellow. **(D)**Case 2 physical examination. **(E)** One grayish-red unshaped tissue measuring 9.5cm×5.5cm×4.2cm. **(F)** The cut plane of the tumor is partially grayish red jelly-like and partially grayish yellow soft in texture.

The left side of the scrotum was enlarged, hard, without tenderness, with poorly defined borders, measuring about 15 cm×10 cm, and the mass extended to the left inguinal region. The left testis and epididymis could not be palpated. The right testis and epididymis were well developed. The right testicle and epididymis were developed. The transillumination test was negative. (**Figure 1A**).

#### Laboratory examinations:

AFP 0ng/mL, HCG 0mIU/mL, LDH 171U/L.

#### Imaging examinations:

**Figure 2:**
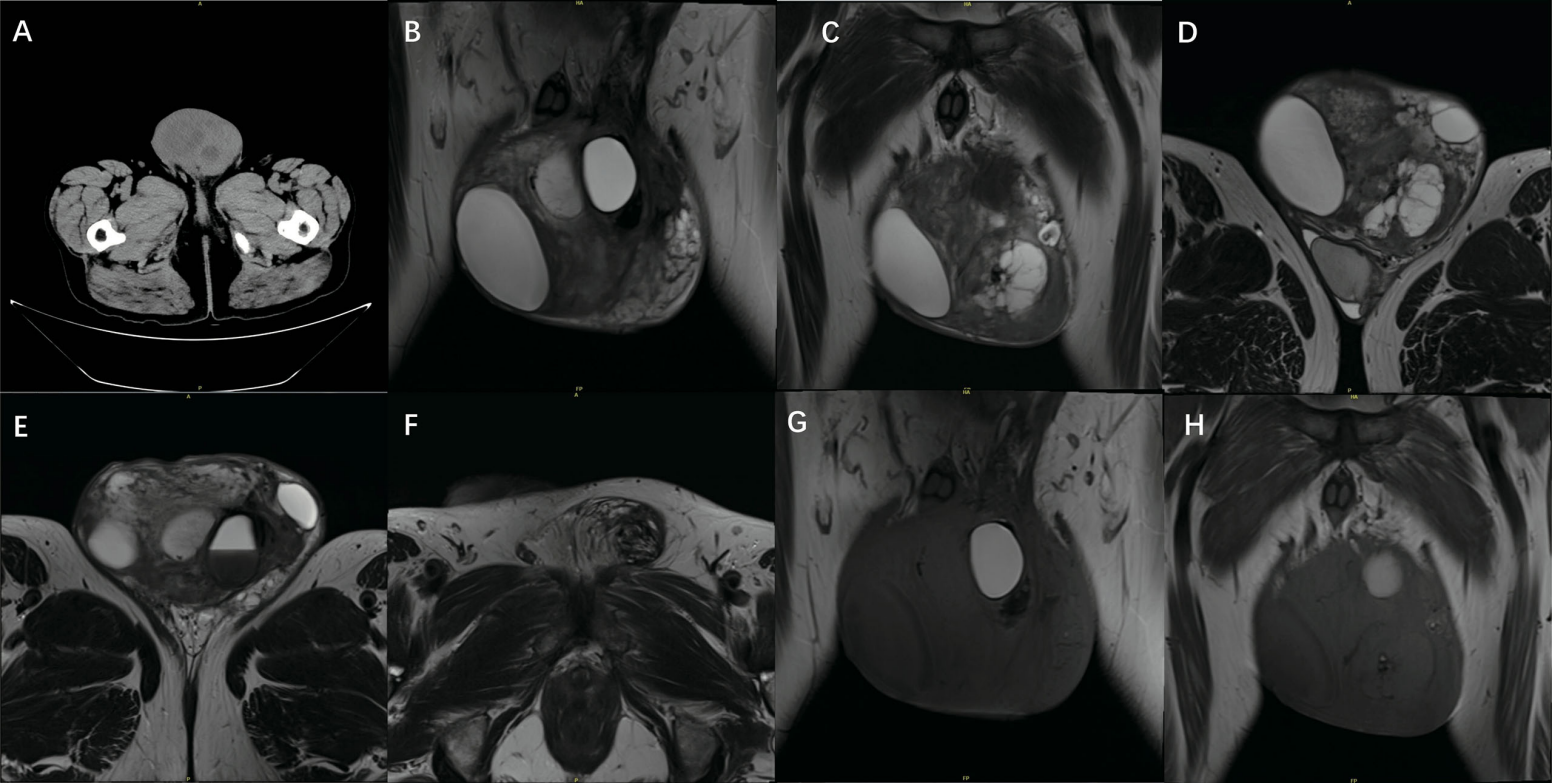
CT: **(A)**: CT shows heterogeneous isometric, low-mixed tissue mass foci in the scrotum. MRI: **(B)**: The left testicle is visible in the T2 coronal position. **(C)**: T2 coronal view shows tumor tissue **(D)**: T2 axial view shows tumor parenchyma, and the testis squeezed to the bottom. **(E)**: T2 axial view shows the testis. **(F)**: T2 axial view shows the mass extending to the inguinal region. **(G, H)** T1 coronal view shows tumor NMR signal.

CT scan of the urinary tract showed heterogeneous iso- and hypo-mixed tissue mass foci in the scrotum, and the left inguinal canal was dilated, thickened, and slightly blurred around it. (**Figure 2A**) The pelvic enhancement MRI showed that the left scrotum was enlarged, with a mass-like mixed signal in the range of 12.0x12.5x7.8 cm; most of the foci were iso-signal in T1, patchy long T1 signal, cystic short T1 signal, a high and low mixed signal in T2, some of the cystic foci were stratified, a slightly high signal in DWI, a slightly low signal in ADC, the solid part was obviously enhanced after enhancement, the normal structure of the left testis and epididymis disappeared, the left spermatic cord was thickened, and was obviously enhanced after enhancement. (**Figure 2B–H**).

#### Treatment, outcome and follow-up:

The patient underwent resection of the left scrotal mass and left orchiectomy. Intraoperatively, the left testicle was completely encapsulated by a mass that could not be separated. The mass extended to the left inguinal region and encircled the spermatic cord tissue. The inguinal canal was opened, the mass in the inguinal canal was removed, the spermatic cord was ligated, and the mass on the left side of the scrotum and the left testis were removed entirely. He was discharged after 5 d of surgery and recovered well, and there was no sign of recurrence 5 months after surgery.

#### Pathological examination:

##### Gross view

The swelling was greyish grey-yellow tissue, size 15×9×9 cm with greyish grey-yellow tough texture on the cut surface, with mucus sensation, visible areas of haemorrhage, necrosis, and cystic degeneration (**Figures 1B, C**).

##### Microscopic examination

**Figure 3:**
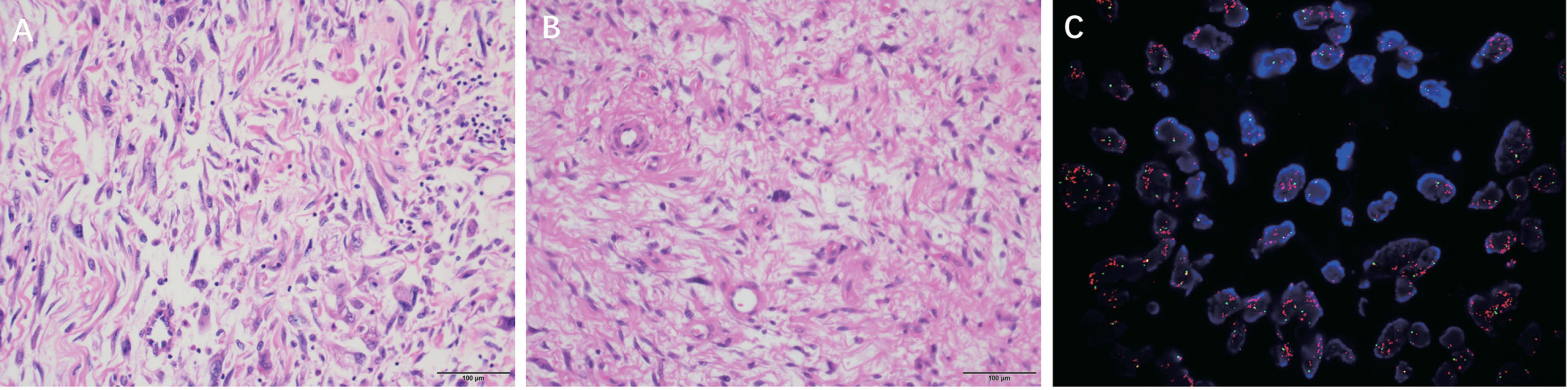
**(A)** DDLPS micrographs showed that the tumor cells in the spindle cell sarcoma area were different in density, and some were arranged in a sclerosing myofibrosarcoma-like arrangement. (H & E, ×200) **(B)** WDLPS micrographs show some atypical lipomatous areas with multinucleated tumor cells and multinucleated rosette-like cells; some are spindle cells and myxoid areas. (H & E, ×200) **(C)** DDLPS MDM2 FISH: MDM2/CSP12: 8.58 MDM2 mean copy number: 20.60.

The mass consisted of many spindle cell sarcoma components and a small number of highly differentiated liposarcoma components, with indistinct demarcation and excessive between the two in some areas. The tumor cells in the area of spindle cell sarcoma were sparse and dense; some of them showed sclerotic myofibrosarcoma-like arrangement, scattered deeply stained mononuclear or multinucleated tumor cells, a prominent proliferation of interstitial collagen fibers or mucus-like changes, a large number of lymphocyte infiltration and lymphoid follicle formation; in some areas, the tumor cells were abundant, with apparent heterogeneity, visible rhabdomyosarcoma-like differentiation, nuclear division like 4~5/10 HPF, and focal pigment phagocyte aggregation(**Figure 3A**).

##### Immunophenotype

**Figure 4:**
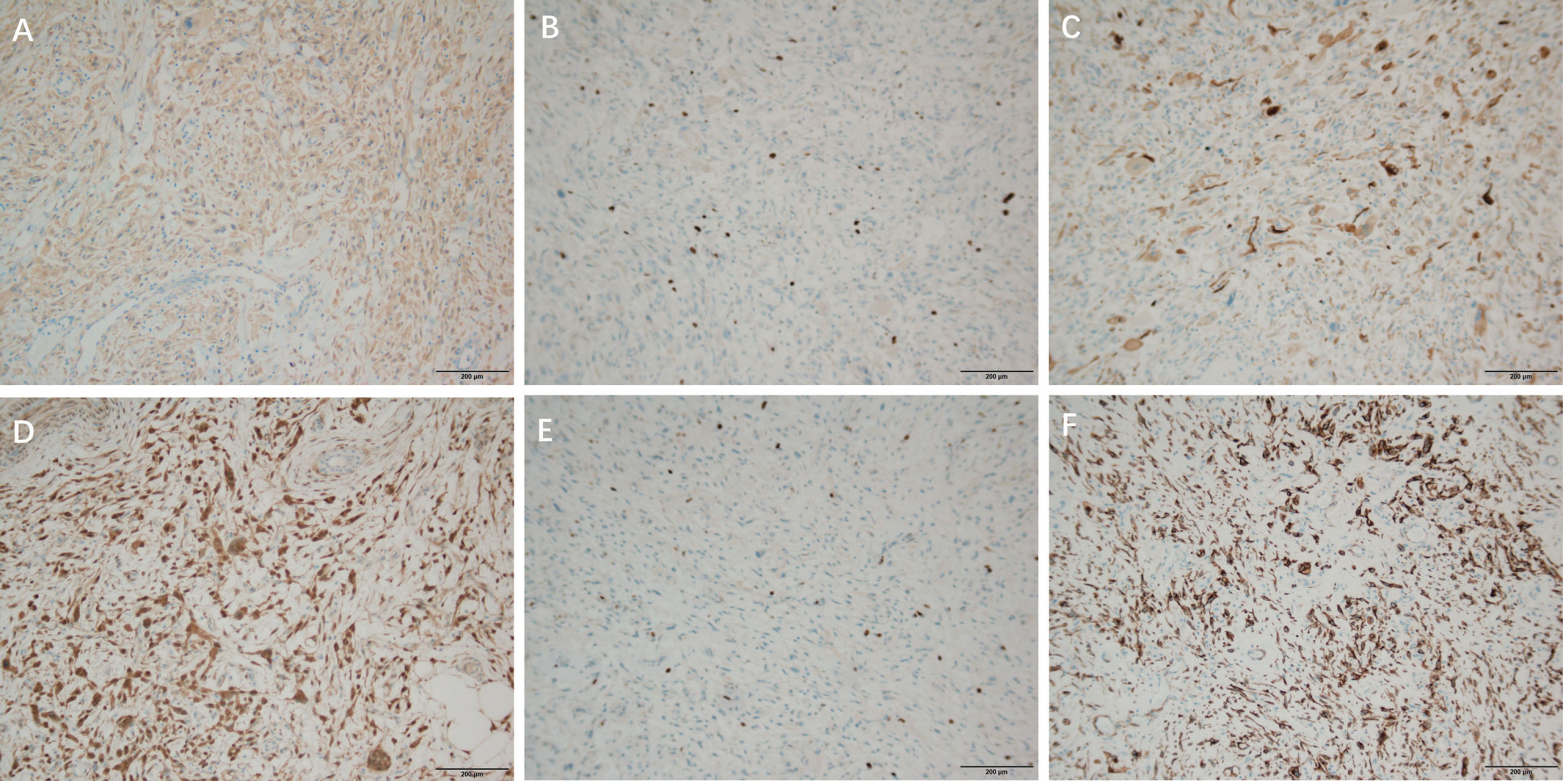
Immunohistochemical staining of DDLPS, ×100 **(A)**: CDK4 (partial +), **(B)**: Ki-67 (proliferation index about 25%), **(C)**: Desmin (2+), Immunohistochemical staining of WDLPS, ×100 **(D)**: CDK4 (-) **(E)**: KI 67 (30% +) **(F)**: Desmin (+).

**Figure 5:**
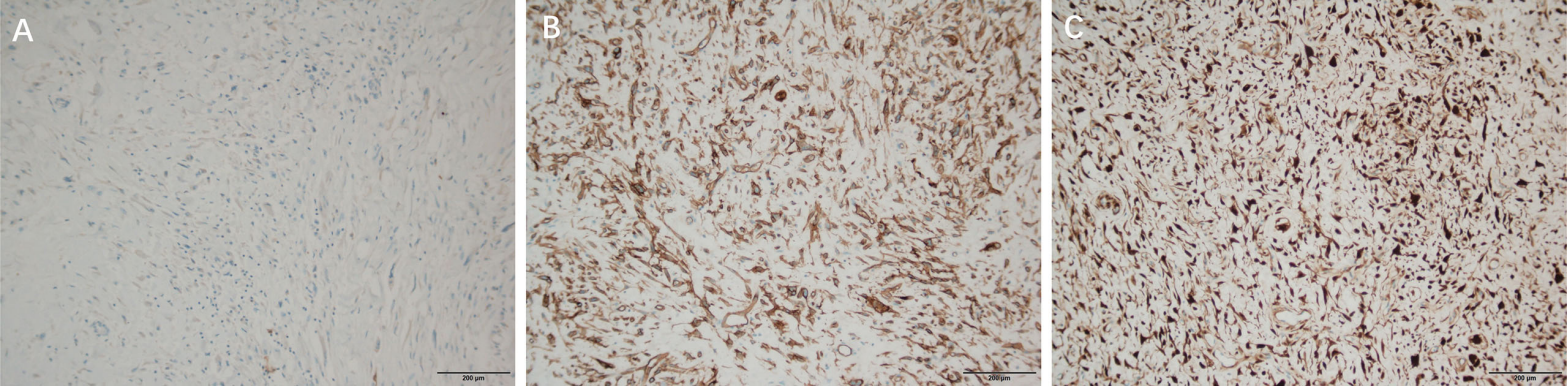
Immunohistochemical staining of DDLPS, ×100 **(A)**: S-100 lipid component Immunohistochemical staining of WDLPS, ×100 **(B)**: CD34 (+) **(C)**: P16 (+).

CDK4 (partial +) (**Figure 4A**), Ki-67 (proliferation index about 25%) (**Figure 4B**), Desmin (2+) (**Figure 4C**), S-100 lipid component (+) (**Figure 5A**), MyoD1 (–), Myogenin (-), CD34 (-), CK (-), SOX10(-).

##### FISH

MDM2 gene amplification (MDM2/CSP12 ratio:8.58) (**Figure 3C**).

#### Pathological diagnosis:

DDLPS

### Case 2:

#### Chief complaints and history of illness:

A 42-years-old male patient was admitted to the hospital with “a left scrotal swelling found for more than six months”. The patient was previously healthy, did not smoke or drink alcohol, and reported no relevant family history.

#### Physical examination:

A hard mass of about 7 cm was palpable in the left scrotum. The testis, epididymis, and the mass were indistinctly demarcated and extended upward to the inguinal region without tenderness.

#### Laboratory examinations:

AFP 2.5ng/mL, HCG 0mIU/mL, LDH 165U/L

#### Imaging examinations:

CT scan shows a mixed, slightly dense shadow in the left scrotum. The enhanced pelvis MRI showed a mass-like mixed signal in the left scrotum. The ultrasound showed an 8.9×5.0×3.1cm hyperechoic area in the left scrotum, with the upper edge reaching the inguinal region and uneven internal echogenicity.

#### Treatment, outcome, and follow-up:

**Figure 6:**
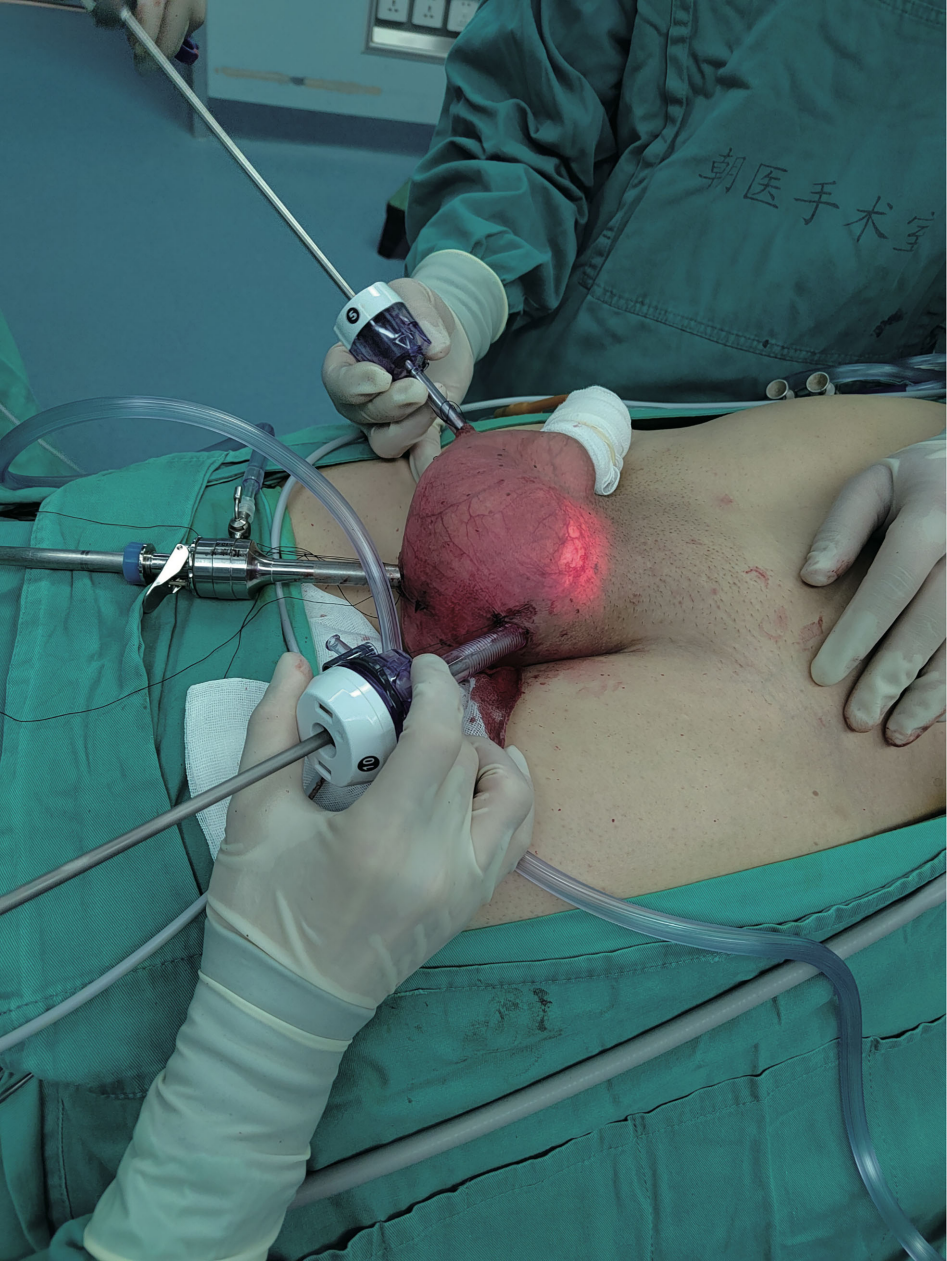
Excision of the scrotal mass with the aid of scrotoscope.

Scrotoscopic excision of the left scrotal mass was performed 6 days after admission (**Figure 6**). Subserosal pneumoperitoneum needle puncture of the left scrotum was performed to connect the pneumoperitoneum with a pressure of 12 mmHg and create a subserosal scrotal cavity. Trocar was punctured at the base of the left side of the scrotum, on the right side of the midline near the root of the penis, and on the left side near the skin. The ultrasonic scalper was used to free the mass from the surrounding sarcolemma and the spermatic cord within the cavity. The mass was seen to be located above the left testis, surrounded by the spermatic cord, with the upper border reaching near the external ring opening. The mass was excised intact, and the spermatic cord was protected. There was no postoperative fever, testicular epididymitis, scrotal hematoma, wound infection, or apparent subcutaneous emphysema, or other complications. He was discharged after 4 days of surgery with good recovery and no sign of recurrence 10 months after surgery.

#### Pathological examination:

Gross view: A grey-red incomplete open tissue, 9.5×5.5×4.2 cm, surface envelope intact, two nodules seen in section, 3.3-6.5 cm in diameter, part of the area grey-red jelly-like, part of grey-yellow soft texture (**Figures 1E, F**).

Microscopic examination: Adipogenic tumor. Some of them showed atypical lipoma-like areas with obvious cellular heterogeneity, abundant cells, multinucleated tumor cells, and multinucleated wreath-like cells: the other part consisted of spindle cells and the mucus-like regions: scattered mononuclear or multinucleated heterogeneous cells, abundant blood vessels, and scattered branching vascular network were seen. Focal ossification might be seen.

Immunohistochemical results: P16 (+) (**Figure 5C**), s100 (individual +), SOX10 (-), CDK4 (-) (**Figure 4D**), KI 67 (30% +) (**Figure 4E**), CD34 (+) (**Figure 5B**), Desmin (+) (**Figure 4F**), CK (-), MyoD1 (-), Myogenin (-).

FISH: Positive MDM2 amplification.

#### Pathological diagnosis:

WDLPS

## Discussion:

The incidence group of PLS is predominantly middle-aged and elderly. Most liposarcoma is located in the pelvic and posterior peritoneal soft tissue spaces, occurring more rarely in the paratesticular region, accounting for 12% of all liposarcomas. PLS may arise de novo from the adipose tissue around the spermatic cord or by malignant transformation of a pre-existing lipoma. It usually presents as a painless, slow-growing inguinal or scrotal soft tissue mass that can grow for a long time without apparent symptoms in the early stages because of the relatively large space for growth. In later stages, pain and accompanying swelling due to soft tissue pulling and compression symptoms appear so that dedifferentiation is primarily present at the time of diagnosis. Moreover, because of its extremely complex anatomical relationship, it is often misdiagnosed as inguinal hernia, lipoma, syringomyelia, sperm cavity, hematoma cavity, epididymitis orchitis, or testicular tumor (3). WDLPS and DDLPS are the most common pathological types of PLS (4). WDLPS has a low metastatic potential and grows slowly, usually with only local recurrence. Some degree of dedifferentiation or transdifferentiation can transform a WDLPS case into a DDLPS with stronger local aggressiveness, local recurrence, potential metastasis, and worse prognosis (1, 5).

CT and enhancement scans are the preferred method for diagnosing PLS. WDLPS tend to have clear margins, a striated or lobular contour, and a predominant fat attenuation with no visible calcification. Internal nodular septations of soft tissue attenuation that demonstrate mild to moderate enhancement help distinguish them from benign lipomas (6). Since DDLPS consists mainly of dedifferentiated components, CT tends to show nonlipomatous high-density masses within, adjacent to, or containing fat masses, similar to the density of skeletal muscle on the flat scan, with necrotic and cystic areas and thick and thin uneven striated separation within the lesion, and significant enhancement and tissue heterogeneity on enhanced scans (7). The CT presentation of this case is consistent with the literature. MRI has higher soft tissue resolution and can be used as a complementary examination to CT to differentiate tumor anatomical relationships and tissue invasion possibilities. Among them, the dedifferentiated component showed equal or slightly low signal in T1WI and mixed high or moderate high signal in the lipid-suppressed phase of T2WI (8). PET/CT helped to identify high-grade liposarcoma, and the sensitivity of diagnosing DDLPS was 83.3%, and the specificity was 83.3% when the SUVmax intercept value was 4 (9).

The differential diagnosis between WDLPS and DDLPS relies mainly on postoperative pathological examination. Histologically, DDLPS is characterized by the coexistence of non-lipogenic sarcomatous areas with highly differentiated areas rich in adipocytes. Multiplex loops or giant rod chromosomes, which consist of amplified fragments of 12q13-15 containing many cancer-related genes associated with tumorigenesis, can be found in DDLPS (10). The most widely studied of these was MDM2, amplified in nearly 100% of patients (11). A series of drugs targeting the MDM2 gene, such as Palbociclib, are under active investigation (12). The application of FISH to detect MDM2 gene amplification is the gold standard for the diagnosis of DDLPS.

Surgical resection is the standard of care for liposarcoma. Still, it remains controversial whether resection should be expanded to include uninvolved organs adjacent to the primary tumor to reduce the likelihood of local recurrence. A study of 83 patients with retroperitoneal WDLPS(RP WDLPS) showed that concomitant organ resection was not associated with a survival benefit in RP WDLPS (13). IKOMA N et al. found that organ invasion rarely occurs in patients with recurrent RP WDLPS, so preservation of unaffected organs should be considered (14). Therefore, we believe that for PLS, local wide excision should be performed when the pathology is confirmed by rapid intraoperative freezing, and radical orchiectomy should be performed at the same time if necessary, and adjacent organs should be selectively removed only when clinically suspected invasion. Regular follow-up after surgery to achieve early detection of tumor recurrence.

We performed the first scrotoscopic scrotal mass excision for PLS in case 2. We found preoperatively that the mass in case 2 had grown along the spermatic cord to the inguinal region. A conventional open procedure would have created an incision approximately 10 cm long in the inguinal region. Moreover, the patient was young and had a strong desire for minimally invasive surgery, so we chose to use the scrotoscopic approach for minimally invasive surgical treatment. After making a small incision in the scrotum, we created a pneumoperitoneum by piercing the trocar needle so that the scrotal tract fitted closely to the trocar needle, thus ensuring a gas-tight seal to keep the CO2 inside the scrotum, and we did not find any CO2 severe leakage during the operation. In case of air leakage, we recommend reinforcing the incision with sutures. The external ring of the inguinal canal was not opened during the operation, which did not lead to gas entering the abdominal cavity through the opening of the external ring of the inguinal canal and forming a peritoneal pneuperitoneum. In the past, scrotoscopy was performed by injecting saline into the scrotum to provide visualization, which was prone to postoperative scrotal edema, but our use of CO2 to dilate the scrotum compensated for this defect (15).

There is still clinical controversy regarding the role of adjuvant radiotherapy or chemotherapy for PLS. In a study that included 265 cases of PLS, adjuvant radiation therapy was found to have no statistical impact on recurrence-free survival (1). Although liposarcoma is less likely to develop distant metastases, chemotherapy may still be required to control the disease in inoperable and metastatic PLS (16). Jones et al. found that dedifferentiated liposarcoma had a response rate of only 25% to first-line chemotherapy (17). A study that included 208 patients with liposarcoma found that an overall objective effectiveness rate of 12% when applying anthracycline-containing chemotherapy (18). Takuro Noguchi et al. reported a giant paratesticular liposarcoma with pulmonary metastases that was well controlled after 1 year of gemcitabine combined with doxorubicin chemotherapy (16). Evidence for effective chemotherapy for PLS is limited, but we believe systemic chemotherapy may contribute to the survival of patients with advanced and distant metastases.

In conclusion, PLS is a rare tumor, which should be differentiated from testicular tumor and external abdominal hernia before surgery. Local wide excision and, if necessary, concurrent radical orchiectomy are the preferred treatments. If the tumor in the scrotum spreads to the groin area, surgical resection with the aid of a scrotoscope can be tried. This procedure avoids the formation of a large incision in the inguinal region compared to traditional open surgery. We will collect more cases later to explore this procedure’s indications and verify its superiority. Margin-positive cases should be re-excised to improve recurrence-free survival. Patients have a higher risk of local recurrence after surgery but less distant metastasis, so long-term follow-up is recommended. Chemotherapy and targeted drug therapy can be tried for patients with existing metastases or inoperable.

## Data availability statement

The raw data supporting the conclusions of this article will be made available by the authors, without undue reservation.

## Ethics statement

Written informed consent was obtained from the individual(s) for the publication of any potentially identifiable images or data included in this article.

## Author contributions

JL composed the manuscripts and literature review. JW and HH provided figures and pathology review. HY and LT had the acquisition, analysis or interpretation of clinical data for the work, revising it critically for important intellectual content and agreement to be accountable for all aspects of the work. All authors contributed to the article and approved the submitted version.

## Acknowledgments

The authors would like to express their gratitude to the pathologist Hongying Zhao of Beijing Chaoyang Hospital, Capital Medical University, who provided the pathological figures.

## Conflict of interest

The authors declare that the research was conducted in the absence of any commercial or financial relationships that could be construed as a potential conflict of interest.

## Publisher’s note

All claims expressed in this article are solely those of the authors and do not necessarily represent those of their affiliated organizations, or those of the publisher, the editors and the reviewers. Any product that may be evaluated in this article, or claim that may be made by its manufacturer, is not guaranteed or endorsed by the publisher.
